# The role of habit in compulsivity

**DOI:** 10.1016/j.euroneuro.2015.12.033

**Published:** 2016-05

**Authors:** Claire M. Gillan, Trevor W. Robbins, Barbara J. Sahakian, Odile A. van den Heuvel, Guido van Wingen

**Affiliations:** a Department of Psychology, New York University, 6 Washington Place, New York, NY 10003, USA; b Department of Psychology, University of Cambridge, Cambridge, United Kingdom; c Behavioural and Clinical Neuroscience Institute, University of Cambridge, Cambridge, United Kingdom; d Department of Psychiatry, University of Cambridge, Cambridge, United Kingdom; e Department of Psychiatry, VU University Medical Center, Amsterdam, The Netherlands; f Department of Anatomy & Neurosciences, VU University Medical Center, Amsterdam, The Netherlands; g The OCD Team, Haukeland University Hospital, Bergen, Norway; h Department of Psychiatry, Academic Medical Center, University of Amsterdam, Amsterdam, The Netherlands

**Keywords:** Compulsivity, Habit, Goal-directed, OCD, Learning

## Abstract

Compulsivity has been recently characterized as a manifestation of an imbalance between the brain׳s goal-directed and habit-learning systems. Habits are perhaps the most fundamental building block of animal learning, and it is therefore unsurprising that there are multiple ways in which the development and execution of habits can be promoted/discouraged. Delineating these neurocognitive routes may be critical to understanding if and how habits contribute to the many faces of compulsivity observed across a range of psychiatric disorders. In this review, we distinguish the contribution of excessive stimulus-response habit learning from that of deficient goal-directed control over action and response inhibition, and discuss the role of stress and anxiety as likely contributors to the transition from goal-directed action to habit. To this end, behavioural, pharmacological, neurobiological and clinical evidence are synthesised and a hypothesis is formulated to capture how habits fit into a model of compulsivity as a trans-diagnostic psychiatric trait.

## 1 Introduction

When we perform an action with some regularity, the associative links between cues in the environment and that action (i.e. stimulus-response links) are strengthened, such that appropriate actions are more easily accessed in the future (Dickinson, 1985). This processes is called habit formation, and it allows us to automate behaviours that do not require planning or organisation. From simple acts like changing gears while driving, to ensembles of actions such as making a series of left and right turns along a familiar route, habits are in theory capable of controlling almost every level of behavioural complexity. Because the habit-learning system operates purely on the basis of historical information, i.e. whether or not actions were rewarded in the past (reinforcement learning: RL), in isolation habit learning is not an optimal way to make choices when faced with rapid changes in the environment. Fortunately, our brains are equipped with a so-called ‘goal-directed’ system that exerts control over habits in light of new information, including changes in the desirability of outcomes (e.g. not seeking food when satiated) and changes to the contingency between actions and outcomes (e.g. when we need to turn a key in the opposite direction to what is typical to unlock a door). The balance between these systems is susceptible to disruption by a range of factors, of which several have been well characterized: those include over-training (Adams, 1982), stress (Schwabe and Wolf, 2009), associative learning conflict (de Wit et al., 2009), working memory demands (Otto et al., 2013a), and the correlation between actions and consequent outcomes (Dickinson et al., 1983). A host of basic neuroscience studies have been conducted in this area, converging on the notion that the neural substrates of the goal-directed control system are dissociable from that of the stimulus-response habit system (Dolan and Dayan, 2013). Specifically evidence from studies employing a range of techniques including lesions (Corbit and Balleine, 2003, Yin et al., 2004, Faure et al., 2005) and optogenetics (Gremel and Costa, 2013) in rodents to functional (Valentin et al., 2007, de Wit et al., 2009, Tricomi et al., 2009, Liljeholm et al., 2015) and structural (de Wit et al., 2012b, Voon et al., 2014) imaging in humans have converged on the importance of the caudate nucleus (dorsomedial striatum in rodents; DMS) and medial orbitofrontal cortex (OFC) for goal-directed control over action and the putamen for the gradual build-up of stimulus-response habit links over time.

Habits have become a popular model of compulsivity in the burgeoning field of dimensional psychiatry, in part because of how relatively well characterized the supportive processes are at the neurobiological level. Considered to be a putative trans-diagnostic trait, maladaptive habits have been proposed to play a role in a host of psychiatric disorders including obsessive–compulsive disorder (OCD) (Graybiel and Rauch, 2000, Gillan and Robbins, 2014), addiction (Everitt et al., 2001), eating disorders (Godier and Park, 2014), schizophrenia (Morris et al., 2014), Tourette׳s syndrome (Groenewegen et al., 2003), and social anxiety disorder (Alvares et al., 2014). This article critically evaluates the hypothesis that habits are a viable trans-diagnostic model of compulsivity. This hypothesis, in our opinion, rests on two assumptions. The first is that the link between habits and compulsions should be *generalisable*, i.e. be relevant for understanding multiple disorders that are clinically characterized by compulsivity, which we define broadly here as a loss of control over goal-directed behaviour. Secondly, and of equal importance, is the assumption that that disruptions in habit learning should be *specific*, i.e. irrelevant to disorders that are not clinically characterized by compulsivity. In the course of this analysis, we will dissect the possibility that excessive habit-learning, although a common feature of many psychiatric disorders, might arise from contributions of distinct neurocognitive deficits. These putative multiple routes to a habit include the contributions of stress, response inhibition and the direct effects of repeated exposure to primary reinforcers like food and drugs. Moreover, we will assess the extent to which there is evidence that these ‘routes’ differentially affect goal-directed (action-outcome: A-O) and habit (stimulus-response: S-R) learning. Finally, we will discuss the possible implications of these ideas for a future of individually tailored treatment assignment.

## 2 Excessive habits or deficient goal-directed control in compulsivity?

The dominance of habitual associations may derive from the incremental acquisition of stimulus-response links over time, but can also be explained by a diminution of goal-directed, action-outcome control over action. In other words, habits (e.g. a lack of behavioural sensitivity to devaluation, see Box 1) can manifest when stimulus-response links become very strong, but also when there is a reduction in our ability to exert control over habits-processes that have dissociable neural substrates outlined above. Behavioural output can perhaps thus be seen as being governed by a mixture of, and even competition amongst, the appropriate A-O and S-R representations. This distinction may be critical to appropriately characterizing certain neuropsychiatric disorders where there may be (1) core deficits in goal-directed behaviour, (2) putatively related deficits in top–down inhibitory control, (3) indirect effects of stress on goal-directed learning and (4) direct effects of exposure to primary reinforcers (i.e. food and drugs) strengthening the stimulus-response associations and/or compromising goal-directed control.

Probably the earliest disorder for which habit learning was considered a viable model was drug addiction (Robbins and Everitt, 1999, Gerdeman et al., 2003, Everitt and Robbins, 2005, Hogarth et al., 2013). Specifically, the development of compulsive drug-seeking was linked to a transition to habit-based behaviour and a devolution of control over such responding to the dorsolateral striatum (DLS, posterior putamen in humans), that portion known to be implicated in S-R learning (see Dolan and Dayan, 2013 for review). Although different drugs of abuse vary in their pharmacological mechanisms of action, and therefore have differential direct effects on habit learning and goal-directed control, here we focus on their shared rewarding properties which, much like any primary reinforcer, stamp in stimulus-response habitual associations over time. Considerable indirect and direct evidence has since accrued to support the view that drug use is particularly prone to habit-forming: Zapata et al (2010) showed that cocaine-seeking in rats in a two lever ‘seeking-taking’ schedule of administration became insensitive to devaluation of ‘taking’ after a prolonged cocaine taking history. They were also able to demonstrate that inactivation of the DLS reinstated sensitivity of seeking to the devaluation manipulation, so that it again became goal-directed, a result consistent with studies using ingestive reward (Yin et al., 2004, Balleine et al., 2009). Corbit et al. (2012) similarly showed that instrumental alcohol seeking in rats became insensitive to devaluation after 4 weeks of training and also shown to be mirrored by a shift in control from the dorsomedial striatum (DMS) (a key region implicated in goal-directed control; Dolan and Dayan, 2013) to the DLS. Finally, Dickinson et al. (2002) compared alcohol to food rewards in an instrumental learning paradigm and found that when alcohol reinforced behaviour, it was less sensitive to devaluation (i.e. more habitual) compared to when behaviour was reinforced with food. This suggests that alcohol consumption may be particularly susceptible to habit-formation.

Extrapolating the theory of goal-directed action control versus habit-based responding to human drug abusers has been quite fruitful. One of the first examples is that of Sjoerds et al. (2013) who provided behavioural and neuroimaging evidence for over-reliance on habit learning in alcohol-dependent patients. They used the so-called ‘Fabulous Fruit Game’ (de Wit et al., 2009), which requires subjects to learn associations between stimuli, actions and outcomes. After training, participants are presented with two of these outcomes and asked to perform the response that lead to the still valuable outcome, thus probing their knowledge of action-outcome associative learning during training. Abstinent alcohol-dependent subjects underperformed compared to healthy volunteers, indicating deficits in goal-directed associative learning. Moreover, this lack of knowledge of action-outcome links was accompanied by reduced activity of brain areas implicated in goal-directed action (ventromedial prefrontal cortex/mOFC) and increased engagement of those brain areas implicated in habit learning (posterior putamen). In complementary work utilising a computational analysis of goal-directed (so-called ‘model-based’) learning (Daw et al., 2011, Box 2), patients with stimulant dependence also showed deficits in goal-directed control assayed using their trial-by-trial decision-making behaviour (Voon et al., 2015). Finally, Hogarth et al. (2012) investigated the acute effects of alcohol consumption on devaluation sensitivity for food rewards. Although the sample sizes were low in this study and therefore warrant replication, the authors found that participants who were administered alcohol showed less sensitivity to devaluation compared to a placebo control group, in spite of having intact knowledge of task contingencies and a normal reduction of self-reported desire for the reward following devaluation via selective satiety.

In summary, there is convergence across studies in both rodents and humans suggesting that the shift from associative striatum (caudate nucleus) to sensorimotor striatum (posterior putamen), from goals to habits, in drug abuse is likely driven by simultaneous reductions in goal-directed control over action and increases in habit associations that naturally occur with continued reinforcement of drug seeking behaviour. Finally, studies in rodents and humans support the notion that aside from their general reinforcing properties, certain drugs (e.g. alcohol) may also exert a compounding direct pharmacological effect on goal-directed control and/or habit formation (Dickinson et al., 2002, Hogarth et al., 2012).

A separate line of research has recently examined habit learning in the context of an animal model of binge eating disorder (Furlong et al., 2014). Rats were given access to highly palatable condensed milk either continuously or for restricted periods (which promotes bingeing behaviour) before instrumental training for laboratory grain or sucrose reward. Restricted access rats subsequently showed less evidence of reward devaluation in their instrumental responding, i.e. more habits. Interestingly, this correlated with apparently greater changes in neuronal activity in the DLS, consistent with greater control of behaviour by S-R habits, rather than deficits in goal-directed control, which we would expect to be mediated by DMS or prefrontal changes. This result however, has not converged with a study of human patients with binge-eating disorder (Voon et al., 2015). Although these patients show a similar deficit in goal-directed (model-based) control to those described in stimulant abusers above this deficit was associated with changes in volume in the caudate (DMS) and medial orbitofrontal cortex (OFC) of Binge-Eating Disorder (BED) patients, rather than the putamen (DLS). One explanation for this apparent inconsistency is the important distinction one must make between inducing BED-like behaviour in otherwise healthy rats through over-exposure to palatable foods, and studying patients who have a genetic propensity for such behaviour (Javaras et al., 2008). One possibility is that a genetic predisposition towards psychiatric compulsivity is primarily associated with structural and functional abnormalities in goal-directed brain structures, whereas environmental influences such as repeated exposure to drugs and food may operate preferentially on habit-structures like the putamen.

Finally, the largest body of work investing the role of habit in human compulsivity has been conducted in obsessive–compulsive disorder (OCD) (see Gillan and Robbins, 2014 for review), triggered by the observed neurobiological overlaps in terms of the neural substrates of habit formation and the pathophysiology of OCD (Graybiel and Rauch, 2000, Burguière et al., 2015). There are currently five studies in humans that converge on the basic finding that OCD is associated with a shift in balance away from goal-directed control and towards habits, using different experimental paradigms and different patient cohorts. This was first observed using the Fabulous Fruit Game described above, where OCD patients exhibited deficits in their ability to exert control over S-R behaviour in light of the change in the value of outcomes (Gillan et al., 2011). Next, this result was replicated in the avoidance domain, where patients learned to press pedals to avoid receiving unpleasant electric shocks to their wrists (Gillan et al., 2014b). After training, one of their wrists was disconnected from one of the electrical stimulation devices, removing the threat of shock. Critically, this meant that one of the predictive stimuli from training no longer signalled a shock (i.e. the threat was devalued) and thereby rendered that avoidance behaviour redundant. Once again, OCD patients were more likely than controls to continue to perform the response even though this action no longer served a purpose. Importantly, this study established that continued responding was not driven by any failures in task comprehension or any residual (mistaken) beliefs about threat on the part of OCD patients. In fact, there was some suggestion that compulsive habits in OCD might even contribute to the development of irrational fear (see Gillan and Sahakian, 2015 for an elaboration).

Neither of these studies, however, could reveal if these failures to show sensitivity to devaluation in OCD resulted from deficits in goal-directed control or an excessive build-up of S-R habits. Subsequent studies tackled this issue using trial-by-trial decision-making experiments that did not involve S-R repetition, and instead assessed subjects’ ability to make choices that relied on an ability to predict the likely outcomes of their actions (A-O) (Gillan et al., 2014a, Voon et al., 2014). These studies used mathematical and computational modelling, respectively, and together provided convergent evidence that suggested habit biases in OCD could be explained by a selective deficit in A-O goal-directed control over action. This supposition was further tested using functional brain imaging of a large cohort of OCD patients while performing a devaluation probe test (Gillan et al., 2015a). Specifically, patients in this study performed a modified version of the shock-avoidance paradigm described above, and the authors probed if their tendency to form habits was associated with dysfunction in regions that support goal-directed control (caudate, medial orbitofrontal cortex (mOFC)) or S-R learning (putamen). Habits in OCD were associated with dysfunctional hyper-activity in the caudate and to some extent the mOFC, but not the putamen (DLS). Moreover, the self-reported urge to perform habits evident in OCD patients was parametrically associated with the strength of activity in the caudate. Although one cannot infer on the basis of a null effect that excessive S-R links play no role in OCD, there was no evidence in support of this notion. The finding that aberrant caudate hyperactivity was associated with urges to perform habits in our task adds weight to the suggestion that excessive habits in this disorder are most likely a consequence of failures in goal-directed control over action. This notion converges with the broader suggestion that compulsivity as a trans-diagnostic *trait* may be characterized by abnormalities in goal-directed structures and that this may be separable from the (equally important) direct effects of drug/food rewards on stimulus-response habit-learning in the putamen.

However, it must be noted that a more precise neurobiological characterisation of these deficits in OCD and related disorders is needed. Although greater BOLD activity (as observed in the imaging study above) in a given brain region is often intuitively associated with improved performance, we know that this is not the case for OCD. Rather, hyper-activity in the caudate and mOFC are often induced by symptom provocation in OCD (Rauch et al., 1994, Adler et al., 2000, Mataix-Cols et al., 2003, Mataix-Cols et al., 2004, Morgiève et al., 2014) and this hyperactivity is remediated when treatments are successful (Baxter et al., 1992, Swedo et al., 1992, Schwartz et al., 1996, Nakatani et al., 2003, Le Jeune et al., 2010, Zurowski et al., 2012, Figee et al., 2013, Morgiève et al., 2014). So how exactly does hyperactivity produce impaired performance – is this a predisposing factor or a consequence of a lifetime with a compulsive disorder? One way in which researchers have begun to answer this question is through animal models of compulsivity, where one can causally test the relationship between hyperactivity in a given region and compulsive behaviours. Two such studies were recently published using optogenetics in mouse models of compulsivity. The first used a gene mutant model where deletion of a gene (Sapap3) induces excessive grooming behaviour alongside deficits in response inhibition and increased firing of medium spiky neurons in the striatum (Burguiere et al., 2013). Critically, the authors found that they could restore behaviour to the normal range in these mutant mice via compensatory optogenetic excitation of the lateral OFC-striatal pathway, which down-regulated the firing of these striatal neurons. In a complementary study, another group simulated the well-documented hyperactivity in the OFC-ventromedial striatal circuit seen in OCD using chronic optogenetic stimulation. They found that a causal relationship between this hyperactivity and the onset of compulsive grooming behaviour in otherwise healthy mice. Moreover, they found that both grooming behaviour and the hyperactivity induced through chronic optogenetic stimulation were remediated using a common treatment for OCD, the selective-serotonin reuptake inhibitor fluoxetine (Ahmari et al., 2013).

## 3 Response inhibition and habit

In the broadest possible terms, an impaired ability to control behavioural responses is characteristic of both impulsivity and compulsivity (Bari and Robbins, 2013). Impulsive actions are those that are unplanned, prematurely expressed, involve risk and inappropriate to the situation (Daruna and Barnes, 1993). Compulsive actions on the other hand, are characterised primarily as the (sometimes stereotyped) repetition of actions that do not produce valuable outcomes. Although clearly distinguishable, a common feature across these constructs is that in each case behaviour is uncontrolled, and carried out in spite of adverse consequences. That is, whether the response is premature or repetitively executed, both impulsivity and compulsivity reflect a superficially similar lack of executive control over action (Robbins et al., 2012). One possible explanation for this overlap is that failures in response inhibition might, under the right circumstances (perhaps by interacting with other neurocognitive traits), produce both impulsive and compulsive actions. Specifically, in the context of compulsive disorders, failures of response inhibition may contribute to patients׳ inability to exert control over habitual responses. This tenet, which has not been hitherto considered in detail, will be considered here. However we include a caveat that the forthcoming evidence is largely indirect.

Inhibitory performance has been typically assessed using a range of neuropsychological paradigms including those measuring motor response inhibition (e.g. Go/No Go task probing action restraint and Stop Signal task probing action cancellation) and cognitive inhibition (e.g. Stroop task, Flanker task, and Simon task) (see van Velzen et al., 2014 for review). Response inhibition largely relies on a brain network involving mainly right lateralized frontal brain areas, such as inferior frontal gyrus (IFG) and pre-supplementary motor area (pre-SMA), projecting to the motor cortex via the cortico-striatal-thalamic-cortical (CSTC) connections (Chambers et al., 2009). Moreover, the response inhibition networks rely on many neurotransmitter systems, e.g., serotonin, dopamine, noradrenalin, glutamate and GABA. Notably, deficits in inhibitory control have been identified in many neuropsychiatric disorders within a putative impulsive-compulsive spectrum, such as OCD and trichotillomania (Chamberlain et al., 2006), ADHD (Rubia et al., 2011) and addiction (Ersche et al., 2012). This evokes the possibility that response inhibition may contribute to patients’ inability to exert control over prepotent habits. Studies on response inhibition in OCD showed that both interference control and motor inhibition are impaired (van Velzen et al., 2014). Impaired motor response inhibition is also observed in unaffected first-degree relatives of OCD patients (Chamberlain et al., 2007) suggesting that motor response inhibition may be considered an endophenotype for OCD. Also structural (Menzies et al., 2008) and functional (de Wit et al., 2012c) brain correlates of altered response inhibition are present in both OCD patients and their unaffected siblings. Moreover, the reported dysfunction of the inhibition-related network in OCD patients and unaffected siblings seems to be explained, at least partly, by altered connectivity between the IFG and the amygdala (van Velzen et al., 2015) suggesting an important role for the limbic circuit in inhibitory impairment in OCD.

A recent study utilizing the Stroop paradigm in conjunction with a model-based learning task showed that individual differences in cognitive control ability are correlated with the extent to which individuals utilise goal-directed (A-O) learning during decision making (Otto et al., 2014). This suggests that cognitive control and goal-directed control are related psychological constructs. Although it is reasonable to speculate that failures to inhibit a prepotent response might similarly contribute to one׳s ability to control an inappropriate habitual response, a similar comparison of motor inhibition and goal-directed learning is currently lacking in the literature. Indirect evidence for a potential contributory role for response inhibition in the expression of habits in OCD comes from neuroimaging data in OCD, suggesting that abnormal functional connectivity between the rIFG and the mOFC during initial avoidance learning predicts later habit formation in these patients (Gillan et al., 2015a). Future studies should test for a relationship between cognitive and motor inhibition, and goal-directed control in the same patient cohort, and thus test if basic inhibitory deficits can explain the variance in OCD patients’ goal-directed learning performance.

Alternatively, the answer might be even more straight-forward. It is possible that the purported overlap in response inhibition failures across impulsive and compulsive disorders might simply be reflective of the evident flaws in the current psychiatric diagnostic classification system (Robbins et al., 2012, APA, 2013). Given that the assignment of individuals to disorder categories is at best a noisy process and at worst holds no biological relevance, there has been a shift in neuroscience and mental health policy to move towards using Research Domain Criteria (RDoC) when possible (Insel et al., 2013). Another motivation for this change is to improve novel drug development. For example, the treatment of neurobiologically based specific symptoms, such as impulsivity or compulsivity, is likely to be of greater success than attempts to treat heterogeneous diagnostic categories, such as schizophrenia and ADHD (Sahakian, 2014). In theory, an effective treatment for compulsivity could be useful in multiple disorders that share this feature, such as OCD, binge-eaters and drug addicted individuals (Voon et al., 2015).

## 4 Stress, anxiety and habit

Two apparent opposing theories have been postulated to explain compulsivity in OCD. The classic cognitive-behavioural theory of OCD posits that compulsive behaviour is caused by obsessive thoughts. It holds that patients have the irrational belief that their intrusive thoughts could be related to future harm to oneself or others. Having these ideas causes significant distress, which is ‘neutralised’ by performing compulsive actions (Salkovskis, 1985). In a way, this theory implies that compulsions are goal-directed, and in some sense functional, albeit driven by irrational thoughts. However, as outlined in an earlier section, the relationship between obsessions, compulsions and anxiety is now thought to be quite different. Specifically, the recent habit hypothesis of compulsivity posits that compulsive behaviour in OCD results from a deficit in the control over goal-directed actions, leading to increased reliance on habitual actions (Gillan et al., 2011, Gillan and Robbins, 2014). In line with this much more behavioural account of the disorder, OCD has been recently removed from the anxiety disorders section of the DSM, to its own category, ‘obsessive–compulsive and related disorders’ (APA, 2013), reflecting broad consensus that anxiety is not the defining feature of OCD (Bartz and Hollander, 2006).

These opposing models may nonetheless be partially reconciled by considering the influence of stress and anxiety on habitual behaviour. Stress, distress and anxiety are often used alongside or interchangeably. This may not be surprising given the general definition of stress as the nonspecific response of the body to any demand (Selye, 1973), which then also includes the bodily state that is often experienced during anxiety. Clinical observations have long suggested that stress exacerbates compulsive symptoms (Rachman, 1997) and stress also appears to predispose animals to compulsive and inflexible behaviour (Korff and Harvey, 2006). Similarly, initial studies that have investigated the influence of stress on habit behaviour found that a single unavoidable stress procedure already shifts the use from a spatial strategy towards a habitual strategy during a spatial memory task (Kim et al., 2001). This effect appears to be mediated by the neurotransmitter noradrenaline that is released during stress (Packard and Wingard, 2004). Later studies investigated the influence of stress on the relative use of goal-directed and habitual systems using instrumental learning tasks and found that after acute as well as prolonged stress, healthy humans show a marked lack of sensitivity to devaluation (Schwabe and Wolf, 2009, Soares et al., 2012). The effects of stress on promoting habitual behaviour again appear mediated by stress hormones and can be prevented by the administration of an adrenergic receptor antagonist (Schwabe et al., 2010, Schwabe et al., 2011). Rodents are also more likely to display habits after chronic stress, which is associated with hypertrophy of the DLS and atrophy of the prelimbic cortex and DMS (Dias-Ferreira et al., 2009). Similar changes in brain structure occur after prolonged stress in humans. Healthy individuals exposed to a period of prolonged stress have larger putamen and smaller caudate nucleus volumes and show opposite changes in orbitofrontal cortex volume (Soares et al., 2012). Interestingly, the prefrontal cortex is relatively sensitive to acute as well as prolonged stress (Arnsten, 2009, Qin et al., 2009, van Wingen et al., 2012) and changes in cortisol levels are negatively related to goal-directed behaviour particularly in individuals with lower prefrontal cortex capacities (i.e. working memory) (Otto et al., 2013b). This suggests the possibility that stress may primarily reduce prefrontal cortex goal-directed control over behaviour and thereby gives way for predominantly habitual behaviour, which over time may result in neuroplastic changes elsewhere in the brain.

Studies that have investigated the influence of acute stress have also pointed to the role of the amygdala. This brain region is crucial for the detection of salient events and it initiates a cascade of psychological and physiological states that are associated with stress and anxiety (Davis and Whalen, 2001, Ulrich-Lai and Herman, 2009). Furthermore, recent human studies have shown that acute and prolonged stress increase amygdala activity (van Marle et al., 2009, van Wingen et al., 2011). Intra-amygdala infusions of agonists for dopamine or noradrenaline, which are released during stress (Arnsten, 2009), are sufficient to bias behaviour towards the use of habit-like strategies in a water-maze task (Packard et al., 1994, Packard and Wingard, 2004). In addition, while the basolateral region of the amygdala is known to be important for the acquisition of goal-directed actions (Balleine, 2005) recent studies show that the (central nucleus of the) amygdala is mainly critical for the formation of habits by its interaction with the DLS (Lingawi and Balleine, 2012). Together, these studies suggest that high levels of stress (and possibly anxiety) may initially shift goal-directed behaviour towards habitual behaviour by both activating the amygdala and reducing prefrontal cortex control over behaviour, and that preferential habit behaviour may be maintained once structural changes in the frontostriatal system have occurred. However, this hypothesis is largely based on experiments in rodents, and when the accruing evidence from human studies is examined, quite a different picture emerges. Although there is some evidence for basic deficit in fear extinction *recall* in OCD (Milad et al., 2013), this is related to aberrant vmPFC (not amygdala) activity, and in fact fear learning and extinction proceed normally in OCD. Direct investigations of structural and functional neural correlates of goal-directed learning deficits in compulsive disorders have yielded null effects with respect to amygdala involvement, instead highlighting the importance of the caudate and medial OFC (Voon et al., 2014, Gillan et al., 2015a). The idea that compulsivity can be distinguished from the role of fear conditioning in OCD and other disorders at the nexus of compulsivity and anxiety (such as hypochondriasis or body dysmorphic disorder) is further supported by recent work showing that compulsive checking tendencies in OCD that are unrelated to the extent to which stimuli evoke anxiety (Clair et al., 2013). Moreover, in contrast to a fear-based hypothesis of OCD, checking behaviour in general becomes more automatised over time, i.e. requiring less conscious effort (Dek et al., 2015).

Moving beyond the indirect evidence, a critical test of the stress/anxiety hypothesis with respect to goal-directed deficits in OCD is to assess purely anxious patients who do not also suffer from other compulsive-disorders. One recent study attempted to do this and found that patients with social anxiety disorder have a greater tendency to form habits compared to control subjects (Alvares et al., 2014), providing support for a purported relationship between anxiety and habit learning. However, these results must be interpreted with caution as almost all of the patients tested in this study met the criteria for another Axis I disorder, and OCD symptom scores were not collected (and therefore could not be used as a covariate). Therefore, we cannot exclude the possibility that the presumably higher levels of OCD severity in the social anxiety disorder patients (Schneier et al., 1992) drove these effects. If this result is replicable, however, it suggests that perhaps anxiety, which in addition to compulsivity is an important clinical symptom of OCD, may play a role in clinically relevant habit biases in this disorder. This explanation could integrate the classical cognitive-behavioural theory of OCD with the more recent habit account for compulsive behaviour, however more work is needed to directly test this possibility. Future work in particular needs to address the pervasive problem of clinical co-morbidity in psychiatric research. One promising approach in the spirit of a more data-driven approach to psychiatry (Montague et al., 2012, Brodersen et al., 2014) is to conduct large-scale studies that can fully disentangle the presumed dimensional contribution of variation in discrete trans-diagnostic traits to habit-forming tendencies.

## 5 How do habits fit with current treatment models for compulsivity?

We will now discuss if habits as a model of compulsivity have any explanatory power with respect to the efficacy of different treatment strategies. We will largely focus our discussion to OCD, as the relationship between habits and compulsions in this disorder are the most comprehensively characterized to-date. However, given the trans-diagnostic framework advocated here, the implications are in theory generalisable to the treatment of compulsions across many disorders, including substance dependence and binge-eating disorder.

The main pharmacological treatment for OCD are antidepressant drugs, including selective serotonin reuptake inhibitors (SSRIs) (Fineberg et al., 2013). What is the SSRI treatment doing in OCD? One possibility is that serotonergic treatment is reducing rumination and/or anxiety symptoms in patients and thereby indirectly exerting an improvement in OCD symptoms by improving obsessions and anxiety symptoms and thereby indirectly affecting the need to perform compulsions. For example, since high stress induces a tendency to form habits excessively (as discussed earlier) (Schwabe and Wolf, 2009), if the SSRIs are reducing the effects of stress, then the drug may be able to help switch the OCD patient from habitual behaviour towards goal directed behaviour. Along these lines, Faulkner and Deakin (2014) have concluded that the dorsal raphé nucleus projections to the peri-aqueductal grey matter (PAG) restrain panic and escape in anticipation of threat in animals. Robinson et al. (2012), Cools et al. (2008) and Crockett et al. (2009) have all emphasised the effects of serotonin on learning from punishment, but also its impact on reward and resilience. Extending this to OCD, it may be that SSRI treatment is restraining high anxiety levels and reducing the effects of punishment (Morein-Zamir et al., 2013), thereby enhancing the opportunity to benefit from therapies, including exposure treatment aimed at extinguishing maladaptive compulsive habits and irrational fears. This explanation fits with the cognitive theory of OCD described briefly above (Salkovskis, 1985), but suffers from a couple of problems. First, SSRIs are generally prescribed in OCD at much higher doses than is the typically recommended dose for SSRI use in depression and anxiety disorders (Bloch et al., 2010). Second, if the therapeutic effect of OCD is through anxiety reduction, then one would expect benzodiazepines to have a positive effect in OCD, which they do not (Hollander et al., 2003, Bartz and Hollander, 2006).

Another explanation for the therapeutic effect of SSRIs in OCD is based on evidence suggesting that SSRIs are working predominantly in the OFC in OCD patients (el Mansari et al., 1995, El Mansari and Blier, 2006). The OFC, in particular the medial portion, is involved in goal-directed control over action in healthy people (Valentin et al., 2007, de Wit et al., 2009). This region is among the most consistently implicated in OCD (Whiteside et al., 2004), is hyperactive during instrumental learning in these patients (Gillan et al., 2015a), and shows overlapping functional connectivity abnormalities to those seen in addicted individuals at rest (Meunier et al., 2012). In line with the suggestion that serotonergic medication remediates goad-directed deficits in OCD, a recent study has shown that acute typtophan depletion, which reduces serotonin transmission, induces a shift from goal-directed to habitual control over action using the ‘Fabulous Fruit Task’ (Worbe et al., 2015b), and similarly has a deleterious effect of model-based learning (Worbe et al., 2015a). Similar results have been found for reversal learning, which is a task in which a left press might signal food for a series of trials, whereas a right press will not signal any reinforcement. Following a series of successful trials, the contingencies are reversed and the rate of continued responding to the previously rewarded stimulus is measured (perseverance). In marmosets, impairments in reversal learning can be induced by lesions to the OFC (Dias et al., 1996) and the medial striatum (Clarke et al., 2008) and selective 5-HT depletion in the OFC (Clarke et al., 2005). These findings align with recent data examining the impact of SSRIs in OCD found that instrumental learning under both reward and punishment conditions were enhanced in patients receiving SSRI treatment relative to those who were unmedicated (Palminteri et al., 2012), although note this was a convenience sample not a double-blind controlled trial.

Antidepressant treatment in OCD is sometimes augmented by antipsychotic drugs (Denys et al., 2004). Antipsychotics block dopamine receptors and their action as an add-on to antidepressant treatment could be as a major tranquiliser, in an attempt to block stereotyped and ritualised behaviour, or to dampen down the habit system. These latter two possibilities are likely related. For example, antipsychotics are used in the treatment of autism for stereotyped or self-injurious behaviour. The efficacy of antipsychotic medication in treating psychotic symptoms in schizophrenia is directly related to antagonistic effects of these drugs on D_2_ receptors in the striatum. The role of dopamine in compulsive habits has been compounded by studies in rodents; habits that were induced through the rodent model of binge-eating described earlier (Furlong et al., 2014) were eliminated (i.e. goal-directed behaviour was restored) after either dopamine D1 receptor antagonism or an AMPA antagonist infused intra-DLS. This is consistent with previous findings of a role for nigrostriatal dopamine in habit formation in the rat (Faure et al., 2005) and that a regime of amphetamine sensitization (which presumably enhances striatal dopaminergic function) enhances the shift to habits (Nelson and Killcross, 2006). Basic research in humans has produced findings that do not align neatly with studies in rodents, however. For example, a natural link to extending these findings to human neuropsychiatric disorders would concern Parkinson׳s Disease (PD), which might predict a major deficit in S-R habit learning as a consequence of the preferential depletion of dopamine in the dorsal striatum; however, one attempt to test this found results that indicated it was goal-directed learning that appeared to be impaired in patients with this neurodegenerative disease (Nelson and Killcross, 2006, de Wit et al., 2011). Moreover, model-based learning is in fact enhanced by increasing dopamine levels following levodopa administration (Wunderlich et al., 2012), and is associated with higher levels of presynaptic ventral striatal dopamine (Deserno et al., 2015). Similarly, acute tyrosine (dopamine precursor) depletion impairs goal-directed control on the fabulous fruit task (de Wit et al., 2012a). These inconsistencies may stem from dopamine׳s inverted U-shaped effects on working memory and cognitive control (Cools and D׳Esposito, 2011, van Velzen et al., 2014), perhaps interacting in a confounded manner with the general lack of specificity of these designs, i.e. that both Parkinsonism and systemic levodopa administration are non-specific with respect to action within striatal subregions, e.g. in the former case, dopamine depletion is observed in both the caudate and putamen (Bernheimer et al., 1973). In addition, the issue is further complicated when one considers that impairments in dorsal versus ventral circuits in PD depend on medication status and disease stage (Vriend et al., 2014).

The final treatment we will consider in the context of habit learning in compulsive disorders is Exposure and Response Prevention (ERP) (Meyer, 1966), which works best when used in conjunction with SSRIs in OCD (Foa et al., 2005). In ERP, the patient undergoes (i) symptom provocation via exposure to relevant stimuli or situations and (ii) must resist the urge to perform the compulsive act. This treatment does not only produce a reduction in compulsive responding, but also concurrently causes the urge to respond and obsessive thoughts to dissipate (Foa and Goldstein, 1978). So, for example, if the OCD patient had repetitive thoughts about contamination and compulsively hand-washed, the patient would be exposed to dirt or requested to put their hands in toilet water and then prevented from immediately washing their hands. One way in which the efficacy of this treatment for OCD can be explained is as a systematic breaking down of S-R associative links through repeated response prevention exercises. Hyperactivity in the caudate, which has been linked to deficits in goal-directed behaviour in OCD (Gillan et al., 2015a) is remediated when patients respond to ERP (Baxter et al., 1992), adding neurobiological support to this suggestion. Moreover, in OCD, studies have shown that habits induced in the laboratory are closely tied to subjective urges to respond (Gillan et al., 2014b, Gillan et al., 2015a), mirroring the observed concurrent decline in urge and compulsion observed during ERP. Perhaps relatedly, abstinence in addiction, which also breaks habitual associations between cues and drug-taking behaviour, reliably results in a reduction in craving in these patients (Weddington et al., 1990, McClernon et al., 2005). However, the major problem with both ERP and abstinence is that these therapeutic strategies are not tolerated well by patients.

Future research should aim to develop more tolerable mechanisms for disrupting putative S-R links that are deeply entrenched by the time patients typically present for treatment (Sahakian et al., 2010, Insel and Sahakian, 2012). There can be no doubt that S-R links are strengthened with repetition (Adams, 1982, Tricomi et al., 2009), so regardless of whether excessive S-R or deficit A-O learning is the primary cause of compulsivity, OCD and addictions become more difficult to treat with time. In the early stages of the disorder, cognitive behavioural treatments and pharmacological treatments may prove more effective, but patients will have to use these treatments to learn and relearn adaptive behaviours and effective strategies for reducing and eliminating symptoms in future. That is, early intervention studies will require that patients take an active role in their treatment throughout the lifespan (Sahakian, 2014). Early detection and early treatment to reduce stress-induced biasing of the habit system should improve functional outcome, quality of life and wellbeing in patients suffering from disorders of compulsivity, particular that presents with concomitant anxiety. Ideally, this would be combined with treatments that strengthen goal-directed behaviour and top–down cognitive control strategies for controlling urges. Early detection in childhood, adolescents and young adulthood is crucial. New treatments with novel mechanisms of action are required for the treatment of compulsive disorders. These treatments should be aimed at research domain criteria (RDoC), rather than diagnostic categories (Insel et al., 2013), in order to more effectively link biology to symptomatology.

## 6 Summary and conclusions: Generalisability and specificity

Deficits in goal-directed control and associated over-reliance on habits is a model of compulsivity that demonstrates impressive *generalisability* across psychiatric diagnoses that have features of clinical compulsions, including OCD, addiction and binge-eating disorder (Voon et al., 2015). One question raised by this generalisability is how can we explain why particular habits become crystallised in certain individuals? If these compulsive problems are caused by a common underlying trait, why are some individuals compulsive about food but not drugs, and others by washing their hands but not checking the door? An important point to consider here is that the notion that compulsivity is a quantifiable trans-diagnostic trait relevant for many disorders is not the same as saying that these disorders (or indeed all OCD patients) are entirely the same. Individuals diagnosed with different disorders may have many overlapping symptoms, but by their very definition, they have distinguishable clinical phenotypes. These phenotypic differences may be caused via interactions with other putative trans-diagnostic traits such as anxiety, or by external environmental influences (e.g. exposure to food or drugs). Defining these factors and assessing their diagnostic utility is an important question for future research – while relevant trans-diagnostic traits may be highly relevant in terms of differential treatments for OCD versus addiction for example, whereas distinctions between the particular content of compulsions in OCD may be of lesser importance.

Although generalisability is important, of equal interpretive importance, studies attesting to the *specificity* of this effect to disorders of compulsivity are presently in short supply. One likely explanation for this is the pervasive difficulty associated with publishing null results in science. Notwithstanding, two studies examining goal-directed learning in disorders not typically defined as compulsive, social anxiety disorder (Alvares et al., 2014) and schizophrenia (Morris et al., 2014) have also identified deficits in goal-directed control over action, which raises concerns for considering habits a viable a model of compulsivity. There are some alternative explanations for these effects. Firstly, in the respective studies, either co-morbid disorders were not excluded for (Alvares et al., 2014), or patients nonetheless had greater levels of anxiety, depression and stress compared to controls (Morris et al., 2014). Moreover, OCD symptom severity was not measured in either study, and could therefore have explained the results, particularly as higher rates of OCD have been consistently reported in schizophrenia (Poyurovsky and Koran, 2005) (note schizophrenia is not more prevalent in the OCD population), and social anxiety disorder (Schneier et al., 1992). Future studies are needed to directly assess the issues of specificity and expand on what we know about generalisability (e.g. to other disorders of compulsivity e.g. trichotillomania). Until then, we are left with the disconcerting possibility that goal-directed deficits are ubiquitous in all psychiatric populations. If this is the case, then this model will likely not have predictive power with respect to tailoring treatments on an individual basis.

## Disclosures

BJS consults for Cambridge Cognition, Otsuka, Servier and Lundbeck. She holds a grant from Janssen/J&J. She has share options in Cambridge Cognition.

TWR consults for Cambridge Cognition, E. Lilly, Lundnbeck, Teva, Shire Pharmaceuticals, Otsuka. Research Grants; GSK, Lundbeck, E. Lilly. He receives royalties from Cambridge Cognition (CANTAB). He is an editor at Springer and Elsevier.

OvdH received grant support from PhotoPharmics and speaker׳s honorarium from Lundbeck

CMG and GvW have no disclosures

## Role of the funding source

CMG received salary supported from the Wellcome Trust.

## Box 1 Testing for habits

The dominance of habits over goal-directed associative links has traditionally been assessed using two methods, which have each proved to be amenable to cross-species translation (Balleine and O׳Doherty, 2010). The first, and most widely cited, is outcome devaluation (first described by Adams (1980)), which assesses a key definitional feature of goal-directed control: sensitivity of the response to motivational change. In a typical outcome devaluation methodology, the value of the reinforcer of action is reduced (or, less typically, increased: Declercq and De Houwer, 2008), and the experimenter assesses if the animal׳s behaviour appropriately updates in light of this change. Value can be reduced in many ways, ranging from those affecting internal motivational state, i.e. selective satiation (Tricomi et al., 2009) or taste aversion (Adams, 1980), to the external motivational value of outcomes in the environment, i.e. removing threats (Gillan et al., 2014a, Gillan et al., 2014b) or changing the instructed value of an outcome (Gillan et al., 2011). Another, lesser known, method for testing for habits is contingency degradation, which probes a second definitional feature of goal-directed control-sensitivity of the response to the relationship between actions and outcomes. Typically contingency degradation is achieved by presenting (previously response-contingent) reinforcers in the absence of the response, and testing behavioural sensitivity to this change as above (Dickinson and Balleine, 1994). Problematically, deficits in goal-directed control and enhancements of stimulus-response links make the same prediction about behaviour in devaluation and contingency degradation tests, and as such are limited in what they can tell us about putative mechanisms that support the development of compulsivity. Functional neuroimaging can be helpful in this regard, allowing us to make inferences with respect to mechanisms supporting devaluation sensitivity based on neurobiology of goal-directed actions and habits (Gillan et al., 2015a, Gillan et al., 2015b)

## Box 2 ‘Model-based’ learning and goal-directed control

A recent computational framework was proposed which aimed to characterize the reinforcement learning mechanisms that give rise to goal-directed actions and habits (Daw et al., 2011), they were termed ‘model-based’ and ‘model-free’, respectively. Model-free learning is a construct derived from basic temporal difference learning (Sutton and Barto, 1998), the principle that actions which are rewarded in the past are more likely to be repeated in the future – the so-called ‘law of effect’ (Thorndike, 1911). Because this kind of model-free learning is based exclusively on prior experience, it is intuitive to suppose that variation in this quantity might predict how likely an individual is to develop a habit. Model-based learning, in contrast, describes decisions that are not simply based on which worked in the past, but instead uses a cognitive “map” of the world (Tolman, 1948) to make decisions prospectively. This map is thought to contain information about contingency as well as the value of available outcomes in the environment, which are integrated to arrive at an optimal decision. One obvious advantage of utilizing the model-based system is that behaviour is sensitive to rapid changes in the environment – for example we do not need to actually experience a change in how rewarding an action is following devaluation of the associated reward, we can infer it and used this inference to make a better choice immediately. Critically, rather than relying on a terminal devaluation test, this framework (and the associated tasks: Gläscher et al., 2010; Daw et al., 2011; Lee et al., 2014) can be used to estimate the contribution of these learning modes from trial-by-trial learning and thusly get more powerful estimates of variation in these putative cognitive processes.

The notion that model-based and model-free learning map onto goal-directed control and stimulus-response (S-R) habit learning appropriately was assumed for some time, but was only recently tested. In three separate studies, model-based learning during trial-by-trial choice was found to be predictive to devaluation sensitivity, of both those same associations (Gillan et al., 2015b) and entirely different associations (Friedel et al., 2014). Model-free learning, on the other hand, showed no relationship to devaluation sensitivity in any experiment suggesting that it does not adequately track individual differences in the extent to which stimulus-response links build-up over time. In summary, while model-based learning appears to be an appropriate (and powerful) mode of assessing individual differences in goal-directed learning, model-free learning does not track habit formation in the same way. Future work will be needed to refine and reformulate the computational mechanisms that support S-R learning.
